# Supplementary material for: DNA methylation meta-analysis reveals cellular alterations in psychosis and markers of treatment-resistant schizophrenia
Source: eLife. 2021 Feb 26;10:e58430. doi: 10.7554/eLife.58430 (PMC8009672; doi:10.7554/eLife.58430)
Supplement: Transparent reporting form [file elife-58430-transrepform.docx]

***eLife’s* transparent reporting form**

We encourage authors to provide detailed information *within their submission* to facilitate the interpretation and replication of experiments. Authors can upload supporting documentation to indicate the use of appropriate reporting guidelines for health-related research (see [EQUATOR Network](http://www.equator-network.org/%20)), life science research (see the [BioSharing Information Resource](https://biosharing.org/)), or the [ARRIVE guidelines](http://www.plosbiology.org/article/info:doi/10.1371/journal.pbio.1000412) for reporting work involving animal research. Where applicable, authors should refer to any relevant reporting standards documents in this form.

If you have any questions, please consult our Journal Policies and/or contact us: [editorial@elifesciences.org](mailto:editorial@elifesciences.org).

**Sample-size estimation**

- You should state whether an appropriate sample size was computed when the study was being designed
- You should state the statistical method of sample size computation and any required assumptions
- If no explicit power analysis was used, you should describe how you decided what sample (replicate) size (number) to use

Please outline where this information can be found within the submission (e.g., sections or figure legends), or explain why this information doesn’t apply to your submission:

This study builds extensively upon our previous study of DNA methylation in schizophrenia (Hannon et al. Genome Biology 2016) and represents the largest epigenome-wide association study of psychosis to date, and one of the largest analyses of DNA methylation for any disease. It is a meta-analysis of seven independent schizophrenia case control cohorts (total sample size = 4,483 samples) obtained from clinical groups from around the world. Sample sizes of each individual cohort are specified in the ‘Methods and Materials’ Section under ‘Cohort descriptions’ and Table 1. The overall sample size is provided in the Abstract, Introduction, Results and Discussion. We have used a very stringent, published experiment-wide significance threshold derived empirically by our group to avoid false positives.

**Replicates**

- You should report how often each experiment was performed
- You should include a definition of biological versus technical replication
- The data obtained should be provided and sufficient information should be provided to indicate the number of independent biological and/or technical replicates
- If you encountered any outliers, you should describe how these were handled
- Criteria for exclusion/inclusion of data should be clearly stated
- High-throughput sequence data should be uploaded before submission, with a private link for reviewers provided (these are available from both GEO and ArrayExpress)

Please outline where this information can be found within the submission (e.g., sections or figure legends), or explain why this information doesn’t apply to your submission:

The DNA methylation data for this study was generated using either the Illumina Infinium HumanMethylation450 microarray or the Illumina Infinium HumanMethylationEPIC microarray, which has been shown to generate highly reproducible data. As is standard in epigenetic epidemiological studies, we did not run technical replicates using the arrays and instead used available resources to maximize sample size. The data from the seven cohorts were processed through a standard pipeline described in the Methods (*Genome-wide quantification of DNA methylation*), which states the sample filtering criteria and analysis pipeline. Raw data are publicly available through GEO under accession numbers GSE84727, GSE80417, and GSE147221. All analysis code to accompany the quality control and analysis of the data is provided in our GitHub repository.

**Statistical reporting**

- Statistical analysis methods should be described and justified
- Raw data should be presented in figures whenever informative to do so (typically when N per group is less than 10)
- For each experiment, you should identify the statistical tests used, exact values of N, definitions of center, methods of multiple test correction, and dispersion and precision measures (e.g., mean, median, SD, SEM, confidence intervals; and, for the major substantive results, a measure of effect size (e.g., Pearson's r, Cohen's d)
- Report exact p-values wherever possible alongside the summary statistics and 95% confidence intervals. These should be reported for all key questions and not only when the p-value is less than 0.05.

Please outline where this information can be found within the submission (e.g., sections or figure legends), or explain why this information doesn’t apply to your submission:

Complete details of the statistical analysis, including the precise statistical test (and relevant software functions/packages where appropriate) are provided in the Methods and Materials section under the subheadings *Comparison of derived estimates of cellular composition and tobacco smoking, Within-cohort EWAS analysis, Within-patient EWAS of clozapine prescription, Meta-analysis, Overlap with schizophrenia GWAS loci, Enrichment analyses, Gene ontology analysis.* Effect sizes (typically mean differences between groups) and exact p-values are reported in the results sections, and accompanying results tables also include standard errors.

(For large datasets, or papers with a very large number of statistical tests, you may upload a single table file with tests, Ns, etc., with reference to sections in the manuscript.)

**Group allocation**

- Indicate how samples were allocated into experimental groups (in the case of clinical studies, please specify allocation to treatment method); if randomization was used, please also state if restricted randomization was applied
- Indicate if masking was used during group allocation, data collection and/or data analysis

Please outline where this information can be found within the submission (e.g., sections or figure legends), or explain why this information doesn’t apply to your submission:

Experimental groups were defined using standardized psychiatric diagnostic tools by trained clinicians. Psychosis cases were identified using International Classification of Diseases 10th edition (ICD-10) criteria for a diagnosis of psychosis while schizophrenia cases were ascertained using either ICD-10 and/or the Diagnostic and Statistical Manual for Mental Disorders-IV edition (DSM-IV). For each cohort the specific criteria, including additional exclusion criteria, are described in the cohort descriptions, found in the Methods and Materials. Psychosis/schizophrenia case-control status was established at recruitment, and therefore prior to genomic profiling. During the experiment cases and controls were randomized across processing batches (but organized by cohort) to minimize batch effects on the arrays. All samples were run blinded by the laboratory technician undertaking the experiments. Schizophrenia cases were additionally classified into treatment-resistant schizophrenia and non-treatment resistant schizophrenia based on medication records.

**Additional data files (“source data”)**

- We encourage you to upload relevant additional data files, such as numerical data that are represented as a graph in a figure, or as a summary table
- Where provided, these should be in the most useful format, and they can be uploaded as “Source data” files linked to a main figure or table
- Include model definition files including the full list of parameters used
- Include code used for data analysis (e.g., R, MatLab)
- Avoid stating that data files are “available upon request”

Please indicate the figures or tables for which source data files have been provided:

All R code used to perform the reported analyses is available via GitHub (<https://github.com/ejh243/SCZEWAS/tree/master/Phase2>) and can be used to generate the figures and tables presented in this manuscript directly from the raw data tables.
